# Oral migration of *Dirofilaria repens* after creeping dermatitis

**DOI:** 10.1051/parasite/2020015

**Published:** 2020-03-18

**Authors:** Quentin Hennocq, Aloïs Helary, Alexandre Debelmas, Gentiane Monsel, Amandine Labat, Chloé Bertolus, Coralie Martin, Eric Caumes

**Affiliations:** 1 Assistance Publique – Hôpitaux de Paris, Service de Chirurgie Maxillo-faciale et Stomatologie, Hôpital Universitaire Pitié-Salpêtrière, Université Pierre et Marie Curie Paris 6, Sorbonne Université 75013 Paris France; 2 Assistance Publique – Hôpitaux de Paris, Service des Maladies Infectieuses et Tropicales, Hôpital Universitaire Pitié-Salpêtrière, Sorbonne Université 75013 Paris France; 3 Unité Molécules de Communication et Adaptation des Microorganismes (MCAM, UMR7245), Muséum National d’Histoire Naturelle, CNRS 75231 Paris France

**Keywords:** Dirofilariasis, *Dirofilaria repens*, Creeping dermatitis, Cutaneous larva migrans, Oral nodule

## Abstract

We report an autochthonous case of oral dirofilariasis in a 46-year-old female patient exposed in South-Eastern France. The patient first presented eyelid creeping dermatitis of one-week duration, then a sub-mucosal nodule appeared in the cheek. The entire nodule was removed surgically. Histologically, the nodule appeared as inflammatory tissue in which a worm was seen. The molecular analysis, based on cox1 and 12S sequences, identified *Dirofilaria repens*. Ivermectin treatment was given prior to diagnosis, while taking into consideration the most common causes of creeping dermatitis, but treatment was ineffective. The oral form of dirofilariasis is uncommon and could lead to diagnostic wandering.

## Abstract (fr)

Nous rapportons un cas autochtone de dirofilariose buccale chez une patiente de 46 ans exposée dans le sud-est de la France. La patiente a d’abord présenté une dermatite rampante des paupières d’une durée d’une semaine, puis un nodule sous-muqueux est apparu dans la joue. Le nodule entier a été retiré chirurgicalement. Histologiquement, le nodule est apparu comme un tissu inflammatoire dans lequel un ver a été observé. L’analyse moléculaire, basée sur des séquences de cox1 et 12S, a identifié *Dirofilaria repens*. Le traitement à l’ivermectine a été administré avant le diagnostic tout en tenant compte des causes les plus courantes de la dermatite rampante, mais il était inefficace. La forme orale de la dirofilariose est rare et pourrait conduire à une errance diagnostique.

## Introduction

*Dirofilaria (Nochtiella) repens* Railliet & Henry, 1911 is a filarial nematode of the family Onchocercidae. Infected mosquitoes can transmit *D. repens* both to animals (mostly dogs), and accidentally to humans [7, 11, 18]. In most cases, the parasite is found in subcutaneous nodules or in the ocular conjunctiva, although some lung and tumor-like infections have been reported [3, 15, 16].

Humans are an epidemiological dead end, which means that the parasite is usually not able to develop to the sexually mature adult stage. Exceptionally, and in cases of unusual immunotolerance, mature parasites find a sexual partner and generate microfilariae in humans [9]. Definitive healing follows surgical extraction of the filarial worm.

The majority of human cases in Europe have been described in Mediterranean parts of Italy, France, and Greece, and in some Eastern European countries such as Ukraine, the Russian Federation, and Belarus [5, 17]. More than 3500 human cases have been reported in Europe since 1977 [10]. An increase in human cases has been observed in Europe, first in the southern regions and now in the northern parts [5]. The increasing number of cases of human dirofilariasis, and the appearance of new endemic areas in Europe appears to be linked to global warming and mosquito proliferation, and the increasing number of undiagnosed and travelling dogs [4].

We report here a rare case of oral dirofilariasis confirmed to be due to *D. repens* resulting in a clinical setting of creeping dermatitis.

## Materials and methods

### Case-report

A 46-year-old woman, without relevant past medical history, complained of facial skin symptoms. She reported at day 1 (D1) left cheek edema and then, at the end of the same day, lower left palpebral swelling with appearance of millimetric nodules. She noticed, the day after, migration to the glabella, then at D5 to the upper lid and at D6 to the lower lid. On D6 she reported linear swelling of her lower lip seen by her general practitioner (GP) who decided to refer her to an emergency department. The clinical description and the blurred picture taken by the patient (not shown), corresponded to creeping dermatitis. The patient estimated the maximum migration speed of this creeping dermatitis to be about 6 cm in 24 h. She presented to our infectious diseases department on D9. No lesion was observed at this time. She was given a 200 μg/kg single dose of ivermectin to cover most of the parasitological causes of creeping dermatitis. She then described, from D11, painful and inflammatory edema of the left jugal mucosa, which later became nodular.

Before the diagnosis of dirofilariasis was made, the patient had visited an ophthalmology unit, the emergency departments of two different hospitals, and her GP, leading to misdiagnoses of conjunctivitis, shingles and allergy, respectively.

**Figure 1:**
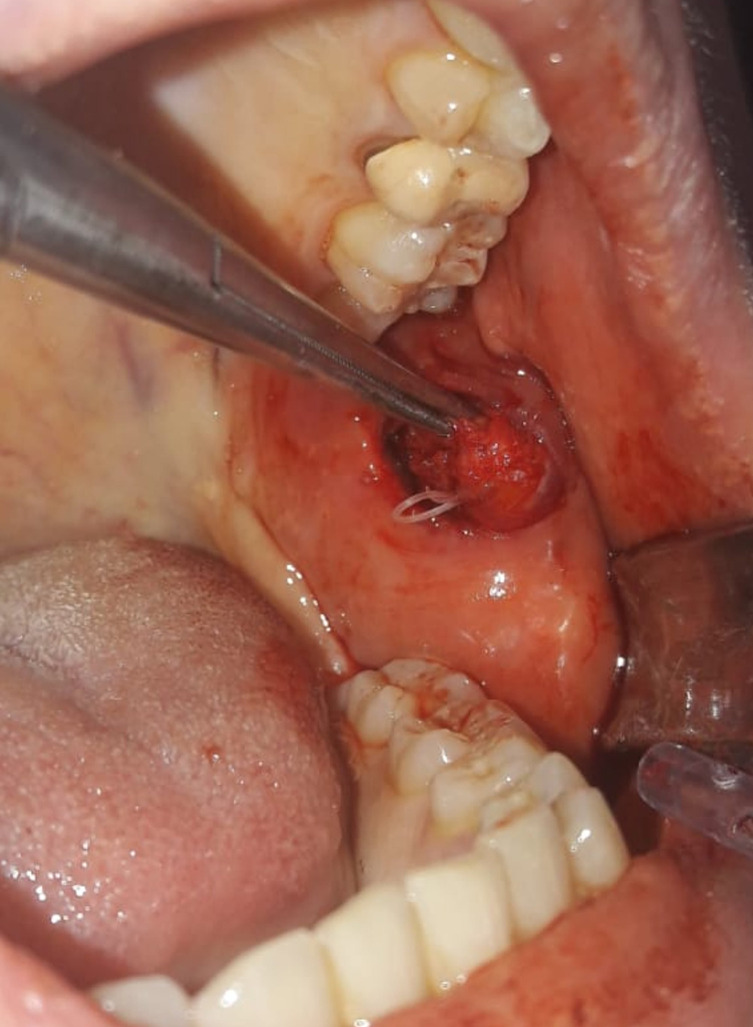
Per-operative photo of the removal of the worm (*Dirofilaria (Nochtiella) repens* Railliet & Henry, 1911) from a sub-mucosal nodule in the cheek.

The entire nodule was removed in the maxillofacial surgery department two weeks later, under local anesthesia of the inner cheek and an inert white worm was observed (Fig. 1).

The patient has always lived in the Paris area. Her last stay outside Paris was in Brittany and the Netherlands six months before, and two weeks in South-Eastern France (Var) in August, which was nine months before. During this last vacation in South-Eastern France, the patient stayed in an urban area in Toulon’s suburbs and reported many mosquito bites. She did not have any hiking activities and was not surrounded by dogs.

### Histologic/microscopic analysis

The nodule was fixed for 48 h by immersion in 4% buffered formaldehyde. The fixed nodule was cut into two halves and both were positioned side by side, embedded into paraffin and 10-μm-thick sections were prepared. The tissues were deparaffinized with toluene and then hydrated using a series of decreasing concentrations of ethanol. Hematoxylin-eosin staining was performed to reveal the general organization of the nodule.

### Molecular analysis

DNA was extracted from a 1 cm median part of the worm using a QIAamp^®^ DNA Mini kit, following the “DNA Purification from Tissues” protocol recommended by the manufacturer (Qiagen, France). PCR products were purified and sequenced by Eurofins Genomics to determine partial sequences of the mitochondrial cytochrome oxidase subunit 1 (cox1), and 12S rRNA genes [13]. The corresponding GenBank accession numbers of other species were used to compare the present specimens using the GenBank^®^ sequence database.

## Results

The blood count showed no eosinophilia, nor inflammatory syndrome. Filariasis serologies were inconclusive. Histologically, the nodule appeared as inflammatory tissue in which the parasite was seen. The lumen content of the nodule consisted of abundant inflammatory cells, predominantly mononuclear lymphocytes, a few mast cells with typical purple-violet granulations, some eosinophils, and rare neutrophils. Some binuclear cells were also observed (Supplementary Fig. 1).

### Morphological analysis

**Figure 2:**
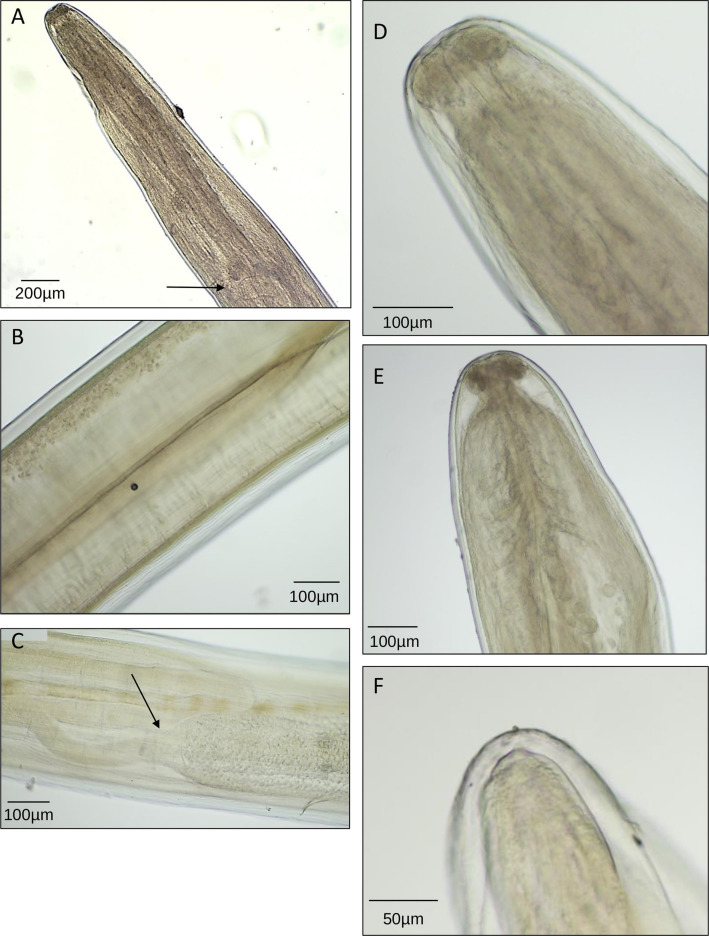
Photographs of the worm, a female *Dirofilaria (Nochtiella) repens* Railliet & Henry, 1911. Characteristics are visible: (A, arrow) vulva opening behind esophagus; (B) empty uteri; (C, arrow) ovary-uterus junction; (D) rounded cephalic extremity and small mouth; (E) short and divided esophagus and lateral deirids with a filamentous structure situated between the nerve ring and esophageal-intestine junction; (F) rounded posterior end.

General: Elongated worm, filiform, with rounded cephalic extremity (Fig. 2D). Short esophagus, divided into muscular and glandular regions (Fig. 2A). Lateral deirids with a filamentous structure situated between nerve ring and esophageal–intestine junction (Fig. 2E). Body surface with longitudinal cuticular crests. Rounded posterior end (Fig. 2F).

Female: Vulva behind esophagus (Fig. 2A). Empty uteri (Fig. 2B).

Measurements: Length 114 mm. Maximum width 450 μm. Esophagus 719 μm long. Vulva 1736 μm to apex.

Morphometric and morphological data are compatible with *Dirofilaria repens*. The worm was an immature female of 114 mm long with a striated cuticle. The absence of oocytes, embryos and microfilaria in the uteri and the ovaries suggests an immature female worm.

### Molecular identification

The DNA sequences of the mitochondrial genes cox1 and 12S rRNA displayed 100% homology with *D. repens.* DNA sequences were uploaded in GenBank under the accession numbers: MT012529 (12S rDNA) and MT012806 (cox1).

## Discussion

This is a rare case of oral dirofilariasis due to *Dirofilaria repens* reported in France [12]. It started with lower eye-lid creeping dermatitis, and ended with a jugal sub-mucosal nodule.

The disease was likely acquired in Southern France (Var), where the patient travelled nine months before, which is not surprising given the increasing prevalence of dirofilariasis in dogs that explains the growing risk of exposure through mosquito bites in this area [4]. Vectors are culicid mosquitoes (*Aedes*, *Anopheles*, *Culex*, etc.). A recent study showed that 1.5% of *Aedes albopictus* – or tiger mosquito – contain DNA from *D. repens* in Corsica [19]. In France, about 100 human cases have been reported since the 1920s, particularly on the Mediterranean coast, Provence, Corsica, and Languedoc, but they are probably underestimated as the benign nature of the cases does not encourage their publication [2]. The parasite seems to be located in the head and neck region in almost 50% of cases, with the periocular region as a privileged site [2, 6].

Dirofilariasis is a rare but identified cause of creeping dermatitis [8]. A study of 74 cases of creeping dermatitis in Paris mainly found hookworm-related cutaneous larva migrans (HrCLM), very few cases of gnathostomiasis, loiasis, and cutaneous pili migrans, whereas there were no cases of dirofilariasis [20]. In Europe, three cases of human dirofilariasis have been reported in the literature with a creeping dermatitis episode: one in France with facial lesions [8], another in Italy with abdominal lesions [9], and the last one in Slovakia with cervical lesions [1]. All the cases developed in a subcutaneous nodule. Only 14 cases of buccal mucosal dirofilariasis have been identified worldwide (France, Bulgaria, Serbia, India, Sri Lanka, Hong Kong, Brazil, USA) [12, 14], with six cases only in Sri Lanka. Specifically, 10 out of the 14 cases were from endemic areas for *D. repens* infection.

Multiple locations of nodules are exceptional in humans infected by *D. repens* and there is usually no microfilaria. As a consequence, surgery alone is the recommended treatment in the vast majority of cases [5, 6] and the use of anthelmintic chemotherapy is not advisable before or after the removal of nodules. Here the patient was treated with a single dose of ivermectin, but the worm was immature and had not produced microfilaria, so the utility of this treatment was limited.

A case of dirofilariasis of the oral mucosa starting as creeping dermatitis was described. These clinical signs can lead to diagnostic wandering. This could be an issue as cases of dirofilariasis are increasing.

## Conflict of interest

All the authors declare that there are no potential, perceived or real conflicts of interest or financial issues related to any commercial associations.

## Supplementary material

Supplementary material is available at https://www.parasite-journal.org/10.1051/parasite/2020015/olm**Supplementary Fig. 1.**
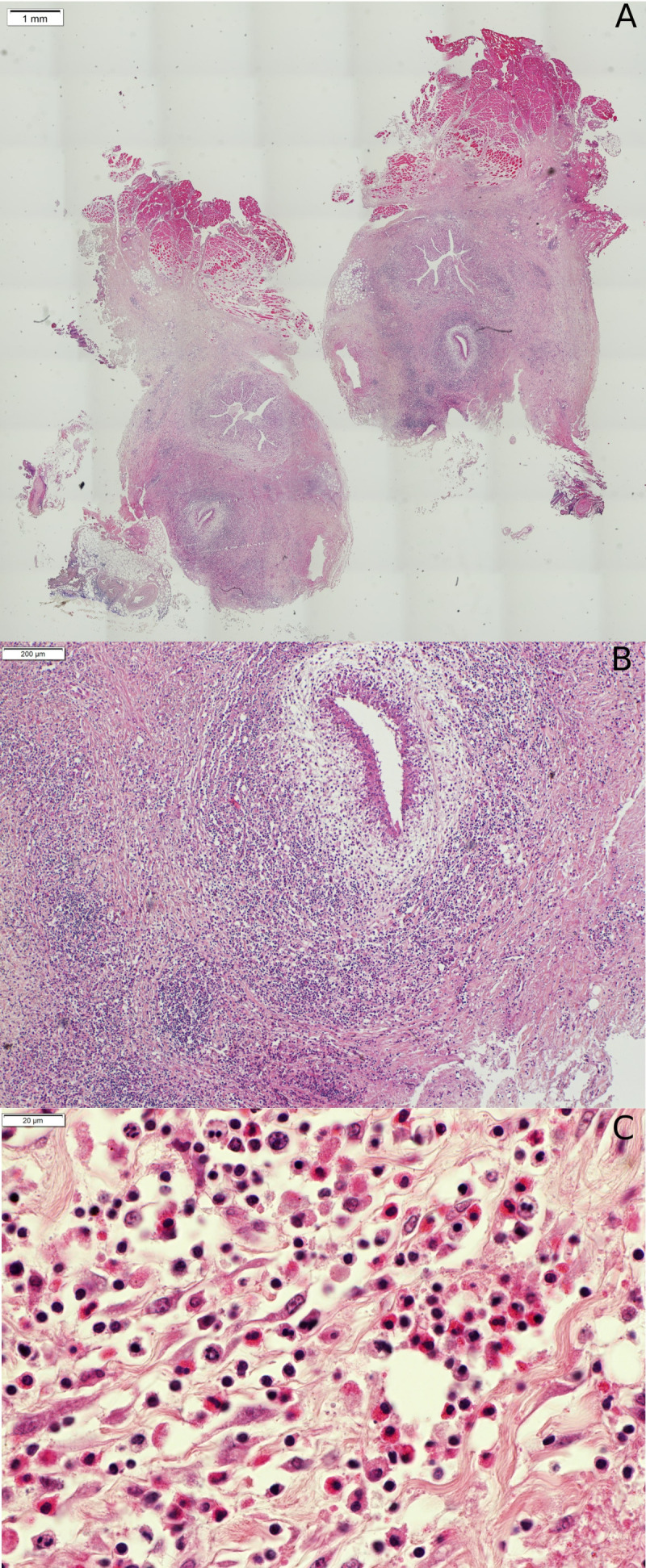
Histological section of the cyst. Sub-mucosal nodule in the cheek with *Dirofilaria (Nochtiella) repens* Railliet & Henry, 1911. (A). A granulomatous immune response (B) is observed with abundant inflammatory mononuclear lymphocytes, a few mast cells typically with purple-violet granulations, some eosinophils, and rare neutrophils. Some binuclear cells can also be observed (C).
